# Pyrrolizidine alkaloid intoxication outbreaks in fattening pigs associated with drought-related feed contamination

**DOI:** 10.1186/s40813-025-00457-2

**Published:** 2025-08-14

**Authors:** M. Leiva-Forns, À. Cobos, L. Martino, S. I. Loscertales, S. Bosco, B. Serrano, A. Rodríguez-Largo, M. Cid-Cañete, N. Valiente, D. Carrión, M. Marcos-Cienfuegos, R. Pagola, J. Martínez, M. Domingo, J. Segalés

**Affiliations:** 1 Servei de Diagnòstic de Patologia Veterinària, Universitat Autònoma de Barcelona, Bellaterra, 08193 Spain; 2 Departament de Sanitat i Anatomia Animals, Universitat Autònoma de Barcelona (UAB), Bellaterra, 08193 Spain; 3 Unitat Mixta d’Investigació IRTA-UAB en Sanitat Animal, Centre de Recerca en Sanitat Animal (CReSA), Campus de la Universitat Autònoma de Barcelona (UAB), Bellaterra, 08193 Spain; 4 IRTA Programa de Sanitat Animal, Centre de Recerca en Sanitat Animal (CReSA), Campus de la Universitat Autònoma de Barcelona (UAB), Bellaterra, 08193 Spain; 5 WOAH Collaborating Center for Research and Control of Emerging and Re-Emerging Pig Diseases (IRTA-CReSA), Bellaterra, 08193 Spain; 6 Cargill Animal Nutrition & Health, Mequinenza, Spain; 7 MSD Animal Health, Salamanca, Spain; 8 Departamento de Sanidad Animal, Universidad Complutense de Madrid (UCM), Madrid, 28040 Spain

**Keywords:** Pyrrolizidine alkaloids, Swine, Intoxication, *Heliotropium europaeum*, Hepatotoxicity, Barley contamination, Climatic conditions

## Abstract

**Background:**

Pyrrolizidine alkaloid (PA) intoxication is a well-documented condition in livestock, resulting from the ingestion of forage and grain contaminated with PA-producing plants. These phytotoxins primarily affect the liver and can lead to severe clinical and pathological disorders, particularly in highly susceptibility species such as pigs. Although sporadic cases of chronic PA toxicosis have been reported in swine, extensive outbreaks affecting large geographic areas have not been previously documented. This report describes a large-scale PA intoxication event affecting multiple intensive fattening pig farms in central Spain.

**Case presentation:**

Between September and December 2023, 21 pig production companies, representing more than 200,000 fattening pigs in *Castilla y León* and *Castilla-La Mancha* autonomous communities (central Spain), reported up to 80% of animals showing prostration, apathy and, occasionally, dark-coloured urine. Mortality during this period ranged 20–40% of affected pigs. At necropsy, animals exhibited variable discoloration of the livers and bleeding gastroesophageal ulcers. Microscopically, hepatic lobes showed an intense interstitial fibrosis and hepatocyte changes including megalocytosis, karyomegaly and canalicular cholestasis. These findings were compatible with chronic toxic hepatopathy. Toxicological analyses ruled out mycotoxins, heavy metals, and pesticides. However, PA contamination was confirmed in a high proportion of compound feed samples associated with contaminated barley with Europine-N-oxide, Heliotrine-N-oxide, and Lasiocarpine-N-oxide. Preventive measures such as changing the source of cereals, reformulating the feed, and using a toxin binder and detoxifying additives allowed resolution of the outbreak.

**Conclusions:**

This report documents a large-scale outbreak of PA intoxication in swine, associated with the use of barley contaminated with PA-producing plants, most likely *Heliotropium europaeum*, in central Spain. Environmental factors, such as drought followed by humid conditions and reduced herbicide application, likely facilitated PA-containing weed growth and subsequent contamination of cereal crops. This case underscores the growing risk of toxicoses linked to climatic and agronomic factors, emphasizing the need for enhanced monitoring and control of feed sources.

**Supplementary Information:**

The online version contains supplementary material available at 10.1186/s40813-025-00457-2.

Pyrrolizidine alkaloids (PAs) are phytotoxins used as a defence mechanism against herbivores by angiosperm plants of the families *Boraginaceae*, *Asteraceae* and *Fabaceae* [1, 2]. About half of the more than 660 PAs and N-oxide derivatives identified in over 6000 plant species exhibit toxic activities [3–5]. Many PAs are frequently encountered as protoxins 1,2-dehydropyrrolizidine ester alkaloids, and the corresponding N-oxides [1, 2, 6]. These protoxins require metabolic activation via cytochrome P450 monooxygenase enzymes to generate highly reactive dehydropyrrolizidine alkaloids (DHPAs) [2, 5, 7]. DHPAs can bind covalently to amino acids, proteins, and nucleic acids, causing genotoxic and carcinogenic effects [7–9]. The liver is the primary target organ; however, other cells such as proximal convoluted renal tubules and club cells in the lung can also generate metabolites that cause local injury [2, 9].

PA intoxication has been described as a cause of livestock death in different countries [10]. Common clinical signs of chronic PA toxicosis include anorexia, depression, icterus, oedema, and ascites, which may manifest acutely once 80 to 90% of functional liver capacity is lost [11, 12]. Affected livers are reduced in size and show paleness and a firm consistency [11, 13]. The main histologic features indicative of PA toxicosis are marked fibrosis, biliary duct hyperplasia, and hepatocellular karyomegaly and/or megalocytosis [9, 11, 13]. Hepatic encephalopathy secondary to hyperammonaemia induces vacuolization of the grey matter and the presence of Alzheimer type II cells [9, 13]. However, brain macroscopic changes are absent [13].

In farm animals, PAs intoxications occur by ingestion of forage and grain contaminated with leaves, flowers and seeds from PA-containing plants [6, 11, 13]. Toxicity of PAs depends on the animal species, age and sex, as well as the metabolic activity and mitotic phase of the target cells [9, 11]. Additionally, young animals are more susceptible to PAs than adults [9, 11]. Pigs, chicken and turkey are very sensitive to the toxic effects of PAs; in contrast, small ruminants are more resistant, whereas horses and cattle have intermediate susceptibilities [9–11]. Grazing animals use to avoid PA-containing plants due to their unpalatability. However, conditions of overgrazed pastures or drought favour the growth of weeds [6, 11, 13].

In pigs, two chronic PA toxicosis cases due to contaminated feed have been previously reported in Australia, and both occurred in a single piggery. In 1977, *Crotalaria retusa* was found as a seed contaminant in grain used as compound feed [14]. The second case, in 1981, *Heliotropium europaeum* was identified as a seed contaminant in peas in a mixed meal ration for 10–16 weeks old grower pigs [15]. These reports occurred as isolated cases of toxicity, with no documented instances of multiple outbreaks to date. Therefore, the aim of this case report is to describe the clinicopathological findings of a chronic PA poisoning outbreaks affecting multiple intensive farms of fattening pigs in central Spain. The clinical condition occurred after consumption of compound feed containing naturally contaminated barley with PAs.

Between September and December of 2023, swine veterinarians from 21 production companies in central Spain (autonomous communities of *Castilla y León* and *Castilla-La Mancha*), housing more than 200,000 fattening pigs, including crossbreeds of Duroc and Iberian pigs, reported up to 80% of the animals (average body weight of 50–70 kg) showing prostration and apathy with no fever and, occasionally, dark-coloured urine. In the late fattening stages, mortality rates ranged between 20 and 40% depending on the farm.

On-farm necropsies revealed diffuse paleness of skin or icterus, and bleeding gastroesophageal ulcers as the most common findings (Fig. 1A). Almost 50% of necropsied pigs showed an intense orange-to-yellow discoloration of the liver (Fig. 1B and 1) with a notably rough surface and a marked generalized lobular pattern. Based on gastroesophageal ulcers and liver damage, vitamin E deficiency was firstly suspected. Indeed, affected animals had low levels of vitamin E in blood, but feed levels were within normal values. The injection of a vitamin E based product did not improve the condition and, therefore, the dietary deficiency of this vitamin was ruled out as a cause.

**Fig. 1:**
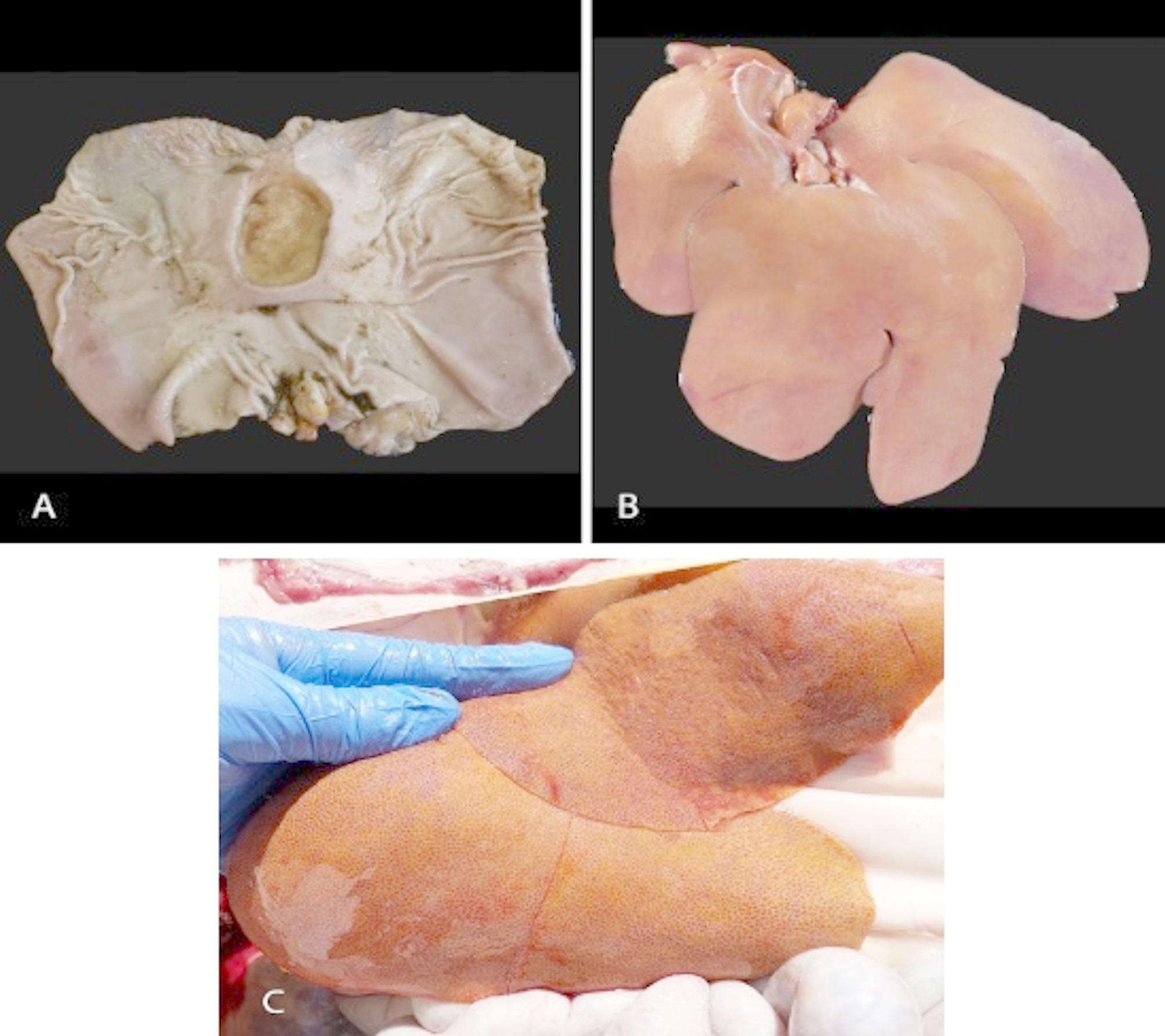
Macroscopic findings in affected pigs. (**A**) Cut stomach by the major curvature revealing an ulcer in the *pars esophagea* region. (**B-C**) Affected livers displayed variable colour ranging from brown-orange to yellow and showed a rough surface

Further necropsies (*n* = 23) from 9 different producers were performed on affected pigs to decipher the cause of these outbreaks and histopathological examination was carried out on collected samples. These included liver (*n* = 23), heart (*n* = 6), kidney (*n* = 5), spleen (*n* = 5), mesenteric lymph node (*n* = 3), whole brain (*n* = 3), lung (*n* = 2), skeletal muscle (*n* = 2), ileum (*n* = 2), superficial inguinal lymph node (*n* = 1), adrenal gland (*n* = 1), urine bladder (*n* = 1) and/or diaphragm (*n* = 1), which were fixed in 10% neutral buffered formalin and sent to the Veterinary Pathology Diagnostic Service (*Facultat de Veterinària*, *Universitat Autònoma de Barcelona*, Spain). Tissue sections were embedded in paraffin, cut into 4 μm sections, stained with haematoxylin and eosin and examined under light microscope.

Microscopically, the most significant lesions were detected in the liver. Hepatic lobules were markedly disrupted by the collapse of sinusoids and an intense interstitial fibrosis (Fig. 2A), which was more pronounced in the periportal region. Remaining hepatocytes showed different changes, including megalocytosis, karyomegaly (Fig. 2B) and abundant nuclear pseudoinclusions (Fig. 2C). Bile canaliculi were markedly distended due to the accumulation of ochre-coloured material (canalicular cholestasis) (Fig. 2D). In addition, ductal proliferation was conspicuous in the periportal area (Fig. 2E). Only brain samples from three animals were submitted and processed; histological findings consistent with hepatic encephalopathy were observed in two of them. In brief, intense vacuolization of the neuroparenchyma (*status spongiosus*) in both white and grey matter (Fig. 3A), affecting the thalamus, pons, cerebral and cerebellar cortices, in decreasing order of severity was found. Furthermore, numerous enlarged astrocytes with loose chromatin and peripheral dispersion (Alzheimer type II astrocytes) were identified (Fig. 3B).

**Fig. 2:**
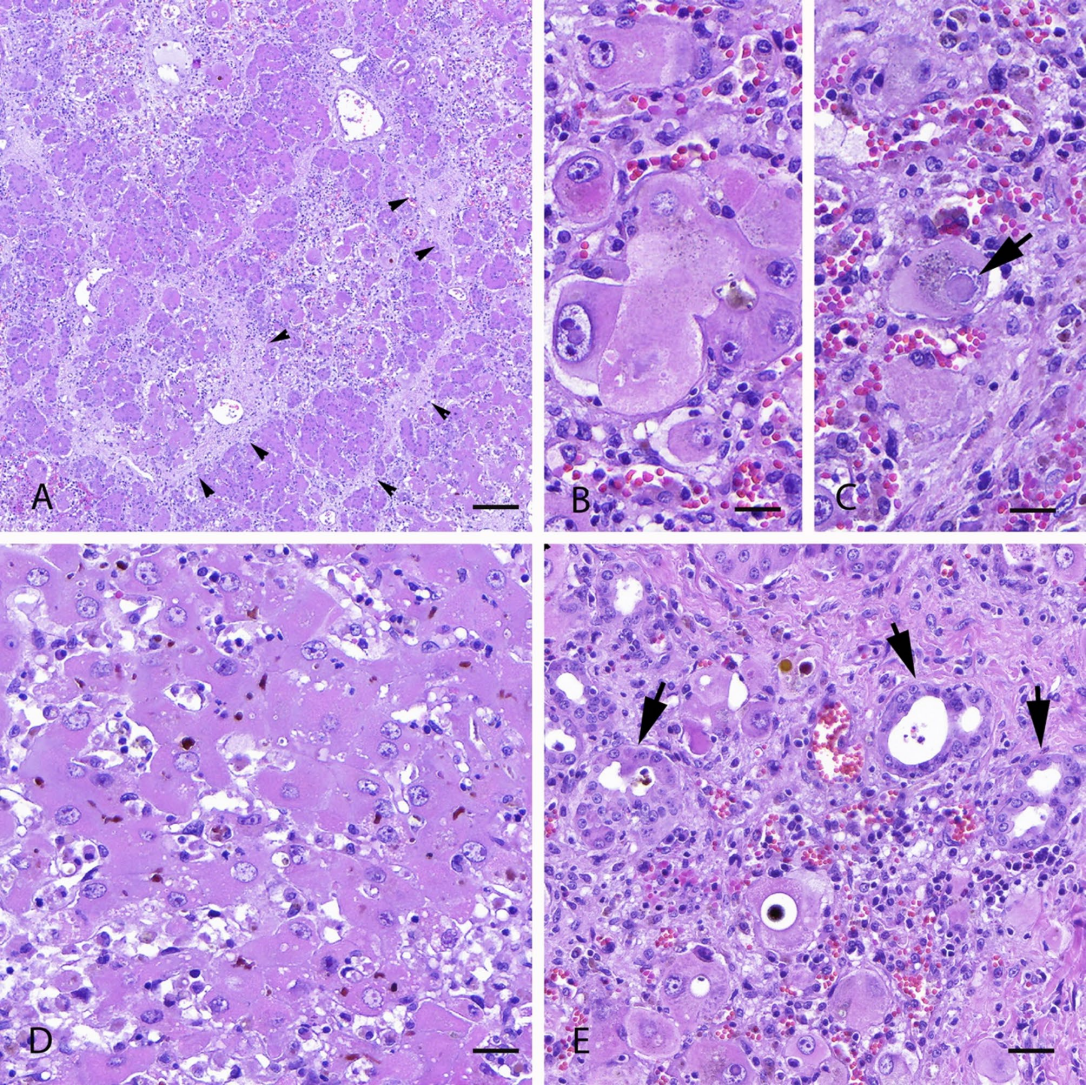
Liver, haematoxylin/eosin stain. (**A**) Loss of normal hepatic architecture with intense interstitial fibrosis (arrowheads) around isles of remaining hepatocytes. Scale bar = 400 μm. (**B**) Megalocytic and karyomegalic hepatocytes. Scale bar = 60 μm. (**C**) Hepatocyte showing a nuclear pseudoinclusion (arrow). Scale bar = 60 μm. (**D**) Canalicular cholestasis with marked distension of bile canaliculi with ochre-coloured material. Scale bar = 80 μm. (**E**) Intense ductular proliferation, with numerous newly formed bile ducts (arrows). Scale bar = 90 μm

**Fig. 3:**
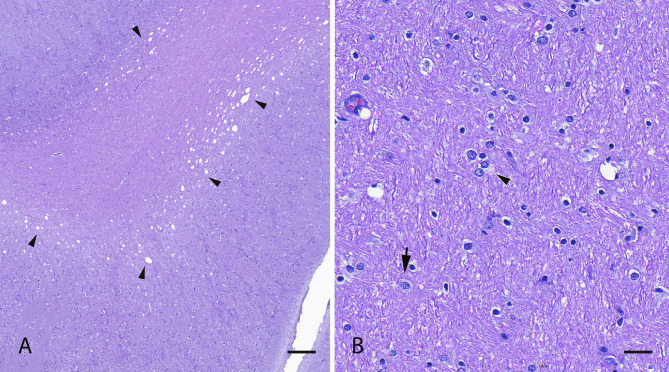
Brain, haematoxylin/eosin stain. (**A**) Vacuolization (arrowheads) of the neuroparenchyma (*status spongiosus*). Scale bar = 1000 μm. (**B**) Presence of numerous Alzheimer type 2 astrocytes (arrows), with loose chromatin and peripheral dispersion, frequently found in pairs or triplets (arrowhead). Scale bar=50µm

Histopathological findings were suggestive of a chronic toxicity process, potentially caused by mycotoxins, pyrrolizidine alkaloids (PA), or oxidative toxins, or less probably by heavy metals and pesticides. Therefore, toxicological tests in tissues (liver and kidney) and feed samples were undertaken.

Fifty compound feed samples were processed by dispersive solid-phase extraction (QuEChERS method) and analysed by combined liquid chromatography with tandem mass spectrometry (LC-MS/MS) for PAs, and results surpassed the detection threshold of the following alkaloids: Heliotrine-N-Oxide (45/50 samples), Europine-N-Oxide (40/50 samples), Lasiocarpine-N-Oxide (36/50 samples), Heliotrine (1/50 samples) and Lasiocarpine (1/50 samples) (Supplementary table). Further investigations into the feed components identified barley as the source of contamination, which represented more than 40% of the compound feed formulation. The detailed concentrations and percentages of the detected compounds in barley samples are summarized in Table 1.

**Table 1:** Pyrrolizidine alkaloid profile of contaminated barley pools from *Castilla-La Mancha* and *Castilla y León*, including concentration of each detected toxic and the percentage that represented over the total concentration (Commission regulation (EC) Nº 1881/2006 of 19 December 2006)

Pyrrolizidine alkaloid	Castilla-La Mancha barley pool (µg/Kg, %)	Castilla y León barley pool - Sample 1 (µg/Kg, %)	Castilla y León barley pool - Sample 2 (µg/Kg, %)
**Europine**	239 (2.8)	184 (1.09)	226 (1.87)
**Europine-N-Oxide**	1,310 (15.1)	3,340 (19.87)	2,470 (20.43)
**Heliotrine**	259 (3)	303 (1.80)	320 (2.65)
**Heliotrine-N-Oxide**	2,790 (32.10)	7,080 (42.12)	5,120 (42.35)
**Lasiocarpine**	287 (3.30)	214 (1.27)	207 (1.71)
**Lasiocarpine-N-Oxide**	3,230 (37.20)	5,550 (33.02)	3,630 (30.02)
**Heliosupine-N-Oxide**	48.8 (0.60)	23.4 (0.14)	22.5 (0.19)
**Rinderine**	42.6 (0.50)	< 20 (0)	< 20 (0)
**Rinderine-N-Oxide**	488 (5.60)	116 (0.69)	94.5 (0.78)
**Total**	8,694.40 (100)	16,810.40 (100)	12,090 (100)

The absence of mycotoxins, including total aflatoxins, deoxynvalenol, fumonisin, ochratoxin and zearalenone, was confirmed through enzyme-linked immunosorbent assay (ELISA). Heavy metals such as arsenic, cadmium, iron, mercury, lead, zinc and copper were analysed using inductively coupled plasma (ICP). Pesticides, including strychnine were assessed by gas chromatography-mass spectrometry (GC-MS), while compounds like 2-aminopyridine, 4-aminopyridine, coumarin, warfarin, coumachlor, dicoumarol, 4-hydroxycoumarin, bromadiolone and coumatetralyl were examined through thin-layer chromatography. No relevant contaminants were found in liver (*n* = 37) and kidney (*n* = 23) samples. Feed samples were also tested for heavy metals through ICP, and 486 pesticides, including different herbicides, fungicides, insecticides, organophosphates, pyrethroids, carbamates, and rodenticides, among others, analysed by gas chromatography and liquid chromatography (GC/LC). Analyses ruled out their involvement in the problem.

After different unsuccessful interventions and based on the results obtained, several preventive measures were implemented to mitigate the impact of the toxicosis. These included limiting the inclusion rate of locally produced barley in all feed formulations, switching to alternative cereal sources, and optimizing feed compositions to minimize the use of regionally sourced barley. In addition, the monitoring of PAs was reinforced in both domestic cereals and compound feed as part of routine quality control procedures. To further reduce the bioavailability of PAs and support hepatic function and recovery, a feed additive package was introduced and maintained until the arrival of the subsequent domestic harvest. This additive contained vegetal charcoal and bentonite, a mycotoxin binder that also exhibit high affinity for PAs. The formulation also included an additive aimed at supporting liver health, composed of carnitine, sorbitol, and various B-group vitamins.

Subsequent analyses confirmed the absence of detectable PAs in the compound feed (Supplementary Table).

Further investigations were conducted to rule out the involvement of common swine infectious agents and other environmental factors. RT-PCR tests for porcine reproductive and respiratory syndrome virus (PRRSV) and PCR tests for porcine circovirus 2 (PCV2) and porcine circovirus 3 (PCV3) were performed, yielding negative results. Additionally, physical, chemical, and microbiological analyses of water sources were conducted to eliminate potential contamination as a contributing factor. These water parameters were within the reference ranges.

The present report describes the occurrence of PA toxicity epizootic outbreaks in pigs fed with national barley in central Spain. While previous cases of PA intoxication in pigs have been reported in Australia, these were isolated incidents linked to contaminated feed [14, 15]. By contrast, the present outbreaks affected multiple farms across a defined region and over an extended period (around 4 months). No previous reports described PA intoxication events at a large-scale in swine or other livestock species.

Histopathological findings were consistent with a chronic toxicosis primarily affecting the liver. However, these findings are not pathognomonic, and extensive toxicological analyses were conducted to exclude other potential aetiologies, including mycotoxins, oxidative agents, heavy metals and pesticides. These tests confirmed the absence of such toxins while detecting high levels of PAs in compound feed samples, aligning with the observed pathological findings and establishing them as the primary etiologic agents.

Barley used in the compound feed was identified as the source of PA contamination. Among the detected alkaloids, Heliotrine-N-oxide, Lasiocarpine-N-oxide and Europine-N-Oxide accounted for the highest proportion of the total PA content across all barley pool samples, ranging from 84.4 to 95.01%, primarily produced by *Heliotropium europaeum* [2]. This predominance together with the known presence of *H. europaeum* in Spain, strongly suggests it as the most likely plant involved in the contamination.

The presence of *H. europaeum* in cereal crops is influenced by environmental conditions. Annual weed species, as *H. europaeum*, thrive in disturbed soils and during periods of drought, which favour their growth over other crops and weeds [16, 17]. Data obtained from the Spanish Meteorological Agency (*Agencia Estatal de Meteorología*, AEMET) [18], demonstrated that 2023 was characterized by exceptionally warm and dry spring months across Spain, particularly in the central and southeastern regions, followed by a humid early summer. These climatic conditions, combined with possible reductions in herbicide application due to the expected bad crop, may have facilitated the proliferation of *H. europaeum* in local barley fields, leading to significant PA contamination of harvested grain.

Although PA-induced hepatotoxicity is well-documented, the experimental toxic doses required to induce chronic intoxication in pigs remain unclear. However, an experimental study with *Crotalaria retusa*, a PA-containing plant, provided insight into dose-response relationships in swine. Pigs fed with diets containing 0.02% or higher concentrations of *C. retusa* seeds developed disease and died or were euthanized between 63 and 107 days post-exposure, with a mean survival of 81 ± 11 days. Lower doses (0.01% and 0.004%) did not cause mortality but led to the development of hepatic and renal megalocytosis [14]. These findings suggest that chronic exposure to low levels of PAs can result in progressive organ damage.

Currently, regulatory limits for PA content in animal feed are focused on dairy products due to concerns about human exposure, with limited data available on acceptable thresholds for livestock feed. The Commission Regulation (European Union) 2020/2040 sets a maximum level of 400 µg/kg of PAs in herbal-based supplements for humans [19]. In comparison, in the present case, the contaminated barley samples in this study had an average total PA concentration of 12,531.6 µg/kg (ranging from 8,694.4 to 16,810.4 µg/kg), far exceeding these regulatory limits.

Histopathological examination of the brain revealed lesions consistent with hepatic encephalopathy, a common feature of chronic liver diseases [13]. It results from the accumulation of neurotoxic metabolites, particularly ammonia, due to liver dysfunction [9]. While the findings supported this condition, no neurological signs were observed during this outbreak. However, in chronic conditions, neurological manifestations may be intermittent or absent due to compensatory mechanisms, and sometimes only non-specific depression and lethargy are evident [9].

*Pars esophagea* ulcers were a common gross finding in affected pigs. While PA intoxication is primarily associated with hepatotoxicity, it remains unclear whether a direct link exists between PA exposure and gastric ulceration. The stress associated with chronic disease, anorexia, potential metabolic disturbances secondary to hepatic dysfunction, or additional dietary factors may contribute to this finding [20, 21]. Further investigations are needed to clarify the role of PAs in gastric ulcer pathogenesis.

In summary, this case report highlights a toxicity event in pigs associated with the use of barley in feed, with *H. europaeum* as the most probable source of PA contamination. The combination of drought conditions and a late heavy rainy season likely facilitated the proliferation of PA-producing plants in barley fields not treated with herbicides. This large scale and persistent toxicity episode emphasizes the increasing risk of similar incidents related with environmental and climate conditions that may become more frequent in the future. Improved monitoring of feed sources, especially considering changing agricultural practices due to climate patterns, is essential to prevent future outbreaks.

## Supplementary Information

Below is the link to the electronic supplementary material.

Supplementary Material 1

## Data Availability

All data generated or analyzed during this study are included in this published article and its supplementary material.

## Abbreviations

AEMET: Agencia estatal de meteorología
DHPA: Dehydropyrrolizidine alkaloid
ELISA: Enzyme–linked immunosorbent assay
GC: Gas chromatography
GC-MS: Gas chromatography–mass spectrometry
ICP: Inductively coupled plasma
LC: Liquid chromatography
PCV: 2–Porcine circovirus 2
PCV: 3–Porcine circovirus 3
PRRSV: Reproductive and respiratory syndrome virus
PA: Pyrrolizidine alkaloid
